# Prolonged estrogen deprivation triggers a broad immunosuppressive phenotype in breast cancer cells

**DOI:** 10.1002/1878-0261.13083

**Published:** 2021-08-29

**Authors:** Daniela Hühn, Pablo Martí‐Rodrigo, Silvana Mouron, Catherine Hansel, Kirsten Tschapalda, Bartlomiej Porebski, Maria Häggblad, Louise Lidemalm, Miguel Quintela‐Fandino, Jordi Carreras‐Puigvert, Oscar Fernandez‐Capetillo

**Affiliations:** ^1^ Science for Life Laboratory Division of Genome Biology Department of Medical Biochemistry and Biophysics Karolinska Institute Stockholm Sweden; ^2^ Breast Cancer Clinical Research Unit Spanish National Cancer Research Centre (CNIO) Madrid Spain; ^3^ Genomic Instability Group Spanish National Cancer Research Centre (CNIO) Madrid Spain

**Keywords:** breast cancer, estrogen receptor, HLA, immunotherapy, inflammation, PD‐L1

## Abstract

Among others, expression levels of programmed cell death 1 ligand 1 (PD‐L1) have been explored as biomarkers of the response to immune checkpoint inhibitors in cancer therapy. Here, we present the results of a chemical screen that interrogated how medically approved drugs influence PD‐L1 expression. As expected, corticosteroids and inhibitors of Janus kinases were among the top PD‐L1 downregulators. In addition, we identified that PD‐L1 expression is induced by antiestrogenic compounds. Transcriptomic analyses indicate that chronic estrogen receptor alpha (ERα) inhibition triggers a broad immunosuppressive program in ER‐positive breast cancer cells, which is subsequent to their growth arrest and involves the activation of multiple immune checkpoints together with the silencing of the antigen‐presenting machinery. Accordingly, estrogen‐deprived MCF7 cells are resistant to T‐cell‐mediated cell killing, in a manner that is independent of PD‐L1, but which is reverted by estradiol. Our study reveals that while antiestrogen therapies efficiently limit the growth of ER‐positive breast cancer cells, they concomitantly trigger a transcriptional program that favors their immune evasion.

## Graphical abstract

Under prolonged hormone therapy, ER^+^ breast cancer cells activate an inflammatory transcriptional program, which includes a generalized upregulation of immune checkpoint mediators together with the downregulation of the antigen‐presenting machinery. These findings reveal that, while hormone therapies efficiently arrest the growth of ER^+^ breast cancer cells, they also promote a phenotype switch that favors their immune evasion.

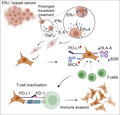

**Abbreviations**
B2M: beta‐2 microglobulin
BC: breast cancer
CAPE: caffeic acid phenethyl ester
CSK: C‐terminal SRC kinase
E2: 17β‐estradiol
EE: 17α‐ethinylestradiol
EMT: epithelial–mesenchymal transition
ERα: estrogen receptor alpha
GSEA: gene set enrichment analysis
HLA: human leukocyte antigen
HTM: high‐throughput microscopy
IFN‐γ: interferon gamma
IL‐6: interleukin‐6
JAK: Janus kinase
MAPK: mitogen‐activated protein kinase
MMTV‐PyMT: mouse mammary tumor virus–polyoma middle tumor antigen
NF‐κB: nuclear factor‐kappa B
PD‐1: programmed cell death protein 1
PD‐L1: programmed cell death 1 ligand 1
PD‐L2: programmed cell death 1 ligand 2
SASP: senescence‐associated secretory phenotype
SERD: selective estrogen receptor degrader
SERM: selective estrogen receptor modulator
SFM: steroid‐free media
STAT: signal transducer and activator of transcription
TNBC: triple‐negative breast cancer
TNF‐α: tumor necrosis factor alpha

## 1 Introduction

Breast cancer (BC) is the most frequent cancer in women worldwide and the second cause of cancer‐related mortality [1]. In around 75% of BC cases, tumor cells express estrogen receptor alpha (ERα) and are dependent on its transcriptional activity for survival and growth [2]. Patients with ERα‐positive tumors (ER^+^, hereafter) usually receive endocrine therapies such as selective ER modulators (SERM, e.g., tamoxifen), selective ER degraders (SERD, e.g., fulvestrant), or aromatase inhibitors (reviewed in Ref. [3]). Unfortunately, and while hormone therapy is effective in arresting the growth of ER^+^ cancer cells and reducing tumor burden, a substantial number of patients relapse into a metastatic stage of poor prognosis [4]. Thus, there is intensive effort in testing the efficacy of new therapies, to be used alone or in combination with hormone therapy, to reduce the percentage of recurrences and improve overall survival. In this context, targeting immune checkpoints by blocking programmed cell death 1 (PD‐1) and/or programmed cell death 1 ligand 1 (PD‐L1) is one of the most promising new cancer therapies and has shown to improve progression‐free survival for triple‐negative BC (TNBC) patients [5]. However, initial evidences indicate that ER^+^ tumors are not very responsive to immunotherapy, which among others might be due to a low mutational burden and low numbers of infiltrating lymphocytes [6].

Estrogens, most frequently 17β‐estradiol (E2), have widespread effects on transcription that are to a large extent mediated by binding to two members of the nuclear receptor family, ERα (*ESR1*) and ERβ (*ESR2*). Upon binding to estrogens, these factors homodimerize and bind to target sequences on chromatin where they regulate transcription preferentially at distal enhancers [7, 8]. Besides their well‐known roles in reproductive organs, estrogens have also effects in other tissues including bone, liver, colon, adipose tissue, kidney, skin, and the cardiovascular and central nervous systems [9]. In addition, several lines of evidence indicate a particularly important role of estrogens in suppressing inflammation, which is thought to contribute to the gender‐related differences found in diseases such as multiple sclerosis or rheumatoid arthritis [10, 11]. Interestingly, the anti‐inflammatory effects of estrogens might underlie the lower mortality rates of female patients to COVID‐19 [12], which has led to a clinical trial to explore whether estrogen patches can reduce the severity of the infection [13]. Importantly, this effect of estrogens is also relevant in cancers that have been linked to inflammation such as hepatocellular carcinoma, which is three to five times more frequent in men than in women [14].

Despite its relevance for human disease, how estrogens regulate inflammation is yet not fully understood. Most studies in this regard have focused on a crosstalk between ERα and the transcription factor nuclear factor‐kappa B (NF‐κB), a key regulatory element of inflammatory responses associated with the development, progression, and therapy resistance in cancer [15, 16, 17]. An example of this crosstalk relates to ERα preventing the binding of NF‐κB to its target sites in the interleukin‐6 (IL‐6) promoter, thereby preventing the expression of this key proinflammatory cytokine [18]. Surprisingly, and although the link between estrogen signaling and inflammation has been known for decades, the contribution of this phenomenon in the context of the estrogen deprivation therapy for ER^+^ BC patients remains largely unexplored. We here reveal that while hormone therapy efficiently arrests the growth of epithelial ER^+^ BC cells, it also triggers a broad inflammatory and immunosuppressive transcriptional program that limits their clearance by the immune system.

## 2 Materials and methods

### 2.1 Cell culture, transfection, and chemicals

A549, MCF7, T47D, ZR‐75‐1, HCC1937, and MDA‐MB‐231 were a kind gift from T. Helleday laboratory, and cell line identity was confirmed using short‐tandem repeat profiling analysis by ATCC. Except for ZR‐75‐1 and HCC1937, which were cultured in RPMI 1640, all cell lines were cultured in Dulbecco's modified Eagle's medium supplemented with 10% fetal bovine serum and penicillin/streptomycin (100 U·mL^−1^) at 37 °C in a 5% CO_2_ humidified incubator. For experiments requiring hormone depletion, cells were cultured in phenol red‐free DMEM or RPMI 1640 supplemented with 2 mm l‐glutamine and 10% charcoal/dextran stripped fetal bovine serum (Sigma‐Aldrich, St. Louis, MO, USA; F6765), hereafter termed steroid‐free media (SFM). Where indicated, 10 nm 17α‐ethinylestradiol (Sigma‐Aldrich, E4876) was added to SFM. For fulvestrant treatments, cells were cultured in standard DMEM supplemented with 1 µm fulvestrant (ICI 182, 780, Tocris, Bio‐Techne Ltd., Abingdon, UK, 1047) for the indicated number of days. During all treatments, the respective media were exchanged every 4 days. For siRNA transfections, cells were seeded in six‐well plates and transfected the next day with 30 pmol of *ESR1* (Sigma‐Aldrich, SASI_Hs01_00078592), *ESR2* (Dharmacon, Horizon Discovery, Cambridge, UK, L‐003402‐00‐0005), or Ctrl (Dharmacon, D‐001810‐10‐20) siRNA using RNAiMAX according to the manufacturer's instructions. Transfection was repeated 3 days later, and cells were harvested for analysis at day 6.

### 2.2 High‐throughput screening (HTS)

The chemical compound library was provided by the Chemical Biology Consortium Sweden (CBCS) and contained 4126 pharmacologically active compounds from the following libraries: Prestwick, Tocris mini, Selleck tool compounds, Selleck known kinase inhibitors, and ENZO tool compounds, as well as 115 covalent drugs synthesized by Henriksson M. (Karolinska Institutet, Sweden). Plate handling and liquid handling were performed using Echo 550 (Labcyte, Beckman Coulter Life Sciences, Indianapolis, IN, USA), Viaflo 384 (Integra Bioscience, Hudson, NH, USA), and MultiFlo FX Multi‐Mode Dispenser (BioTek, Winooski, VT, USA). Images were acquired by IN Cell Analyzer 2200 (GE Healthcare, Milwaukee, WI, USA) with a 10× objective, and quantitative image analyses were run in CellProfiler (https://cellprofiler.org) [19]. Statistical analyses were carried out with Microsoft Excel and graphpad prism software (GraphPad Software Inc., San Diego, CA, USA). For the primary HT screening, A549 cells were trypsinized, resuspended in culture medium containing 100 ng·mL^−1^ human interferon gamma (IFN‐γ; Sigma‐Aldrich), dispensed into 384‐well plates (BD Falcon, Corning, Glendale, AZ, USA, 353962), and incubated for 24 h at 37 °C in a 5% CO_2_ atmosphere. The next day, compounds were added to cells achieving a final concentration of 10 μm and a DMSO volume concentration of 0.1%. Cells were incubated for 24 h before staining with PE‐labeled anti‐human CD274 (Clone MIH1; BD Biosciences, Stockholm, Sweden) antibody. Cells were fixed and stained with 2% formaldehyde and 2 mm Hoechst 33342, respectively. For the validation screening, MCF7 cells were hormone‐stripped by preculturing in SFM for 15 days with several medium changes. Cells were subsequently exposed to DMSO or ethinylestradiol (EE) for 3 days, seeded in 384‐well plates, and incubated overnight at 37 °C in a 5% CO_2_ atmosphere. The next day, the chemical library, comprising 163 compounds, including estrogens and antiestrogens, was added to the cells reaching a final concentration of 0.1, 1.0, and 10 µm. After 72 h of incubation, cells were stained and fixed as described above.

### 2.3 Immunoblotting

Cells were lysed in RIPA buffer (Thermo Fisher Scientific) supplemented with protease and phosphatase inhibitor cocktail (Roche, Sigma‐Aldrich, Stockholm, Sweden), sonicated for 5 min, and centrifuged at 4 °C, at 16 900 ***g*** for 15 min. 50 μg whole‐cell extracts were separated by SDS/PAGE and transferred onto Nitrocellulose membrane (Bio‐Rad). After blocking in 5% milk in TBST, immunodetection was done overnight at 4 °C with antibodies against PD‐L1 (CST, 13684), ERα (CST, 8644), C‐terminal SRC kinase (CSK; CST, 4980), Stat1 (CST, 14994), phospho‐Stat1^Tyr701^ (CST, 9167), p65 (CST, 8242), phospho‐p65^Ser536^ (CST, 3033), p21(CST, 2947), H3K9me3 (Merck, Darmstadt, Germany, 07‐442), β‐actin (Abcam, Cambridge, UK, ab6276), vinculin (Abcam, ab129002), and GAPDH (Millipore Sigma, Sigma‐Aldrich, ab2302). Appropriate HRP‐coupled secondary antibodies diluted in blocking solution were incubated for 1 h at room temperature. Signals were visualized by chemiluminescence (SuperSignal™ West Dura; Thermo Scientific, 34076) and acquired by an Amersham Imager 600 (GE Healthcare).

### 2.4 Flow cytometry

Cells were cultured as indicated and harvested using Accutase (BD Biosciences, 561527). After centrifugation, cells were stained with fluorescently labeled PE anti‐human PD‐L1 antibody (Clone MIH1; BD Biosciences), PE anti‐human PD‐L2, PerCP/Cy5.5 anti‐human β2‐microglobulin, and PerCP/Cy5.5 anti‐human human leukocyte antigen (HLA)‐A, HLA‐B, and HLA‐C (all from BioLegend, San Diego, CA, USA) diluted in 2% FBS‐PBS blocking solution for 45 min at 4 °C. Samples were washed and immediately measured on a Guava easyCyte flow cytometer (EMD Millipore, Darmstadt, Germany). Data were analyzed with Guava InCyte and graphpad prism software (GraphPad Software Inc.).

### 2.5 Quantitative RT‐PCR

Total RNA was isolated using the PureLink RNA Mini Kit (Invitrogen) according to the manufacturer's instructions. Reverse transcription and PCR amplification were performed using TaqMan RNA‐to‐CT 1‐Step Kit and the StepOnePlus™ Real‐Time PCR Instrument (Applied Biosystems, Fisher Scientific, Göteborg, Sweden). The following probes were used in this study: Hs01125301_m1 for *CD274*, Hs99999901_s1 for *18S*, Hs03929097_g1 for *GAPDH*, Hs01046817_m1 for *ESR1*, Hs01100353_m1 for *ESR2*, Hs00174128_m1 for *TNFA*, Hs00989291_m1 for *IFNG,* Hs00174131_m1 for IL‐6, and Hs01058806_g1 for HLA‐A.

### 2.6 Cytokine analysis

MCF7 cells were cultured in different media for indicated number of days, and supernatant culture media were collected every 4 days, centrifuged, and stored at −80 °C until analysis. IL‐6 levels of culture supernatants were determined using the LEGENDplex™ Human Inflammation Panel I (BioLegend, 740809), according to the manufacturer's instructions. Briefly, supernatants (50 μL per sample) were incubated with capture beads for 2 h at room temperature on an orbital shaker. Next, detection antibody was added and beads were incubated for 1 h at room temperature. After washing the beads, samples were measured using a BD LSRFortessa™ flow cytometer (BD Biosciences). Cytokine concentration was calculated based on a standard curve using BioLegend's LEGENDplex™ data analysis software.

### 2.7 Generation of knockout cell lines

For CRISPR/Cas9‐mediated generation of PD‐L1 knockout clones, hybridized oligos (CACCGGCTGCACTAATTGTCTATT) targeting the human *CD274* locus were ligated into the pSpCas9(BB)‐2A‐GFP plasmid (a gift from F. Zhang, Addgene #48138, Teddington, UK) according to standard procedures and transfected into MCF7 cells [20]. GFP‐positive cells were FACS‐sorted, and individual PD‐L1 knockout clones were confirmed by western blotting (WB). For the generation of CSK‐deficient MCF7 cells, the pSpCas9(BB)‐2A‐GFP plasmid was cotransfected with crRNA (target sequence: TACCTTGGTGACGGCCACAA, CM‐003110‐02; Horizon Discovery) according to the manufacturer's protocol. Two days after transfection, GFP‐positive cells were FACS‐sorted into a 96‐well plate and single clones were analyzed for CSK deficiency by WB.

### 2.8 RNA sequencing and data analyses

Next‐generation RNA sequencing was performed to determine changes in gene expression between DMEM (normal)‐, SFM (Treatment 1)‐, fulvestrant (Treatment 2)‐, or SFM+EE (Treatment 3)‐cultured MCF7 cells. Cells were cultured during 20 days in DMEM, SFM, or fulvestrant, and 20 days in SFM with the addition of EE in the last 4 days. The cells were subsequently harvested and frozen at −80 °C, and the samples were processed by Eurofins (Ebersberg, Germany) Genomics Sweden AB, where the RNA was isolated and assessed for QC, and finally, cDNA library preparation was performed. Illumina single‐read sequencing with a read length of 1× 50 bp and 30 million reads per sample was performed.

For gene set enrichment analysis (GSEA), genes in each condition were ranked based on the log_2_ (fold change) value between DMEM (normal) and SFM (Treatment 1) or fulvestrant (Treatment 2). Each treatment was done in triplicate. Genes enriched in each treatment were positive, and genes enriched in normal conditions (DMEM) were negative. The ranked gene lists were loaded into GSEA software and tested against the gene sets of the Hallmark collection (MSigDB).

RNA sequencing and subsequent analyses comparing two different CSK knockout clones with MCF7 WT cells were performed by the Bioinformatics and Expression Analysis core facility of Karolinska Institutet.

RNA sequencing data associated with this work are accessible at the GEO repository, under accession numbers GSE134938 and GSE181909.

### 2.9 T‐cell‐mediated tumor cell killing assay

MCF7 cells were transfected with mCherry‐Nucleus‐7, a gift from Michael Davidson (Addgene plasmid #55110), and a clone was selected. This MCF7 clone was then subjected to SFM, SFM with 10 nm EE, and fulvestrant treatments, as described above. T cells were isolated 5 days prior to addition of MCF7 cells and were activated with Dynabeads® Human T‐Activator CD3/CD28 (Thermo Fisher, 11131D) and 2.5 ng·mL^−1^ of recombinant human IL‐2 (Thermo Fisher, PHC0027). T cells were cocultured with the mCherry‐Nucleus‐7 MCF7 cells in the presence of CellEvent™ Caspase‐3/7 Green Detection Reagent (Thermo Fisher, C10423) in 96‐well plates. Images were captured every 2 hours on a MetaXpress Microscope (Molecular Devices, San Jose, CA, USA). Total MCF7 nuclear count and caspase intensity was analyzed using cellprofiler software.

### 2.10 METABRIC data set analysis

mRNA expression levels of the indicated genes from the BC data set [21, 22] were retrieved from cBioPortal. Only patients with tumor mRNA data were taken into consideration (*n* = 1904). The samples were then classified as ERα^+^ or ERα^−^, and ERα^+^ samples were further subdivided in hormone therapy‐treated or not (− or + HT) using the annotation included in the dataset. The expression levels present in cBioPortal are automatically transformed into *Z*‐scores for comparison purposes. Two‐tailed Student's *t*‐test was used to assess the statistically significant differences in mRNA expression levels of the specified genes between ERα^+^ or ERα^−^ and −HT or +HT patients.

### 2.11 Mouse study

Polyoma middle tumor antigen (PyMT) [FVB/N‐Tg(MMTV‐PyVT)634Mul/J] transgenic animals harboring breast tumors were treated with 4‐hydroxytamoxifen (Sigma‐Aldrich; 1.2 mg·kg^−1^·daily^−1^) in 10% ethanol in sunflower oil by oral gavage. Animals were killed in CO_2_ chamber when tumors reached the humane end point, and the tumors were fixed in 10% formalin solution and embedded in paraffin. For purification of total RNA from formalin‐fixed tumor sections, RNeasy FFPE Kit (Qiagen) was used following the manufacturer’s instructions. Reverse transcription was done using SuperScript™ IV VILOTM Master Mix (Thermo Fisher Scientific), and the real‐time PCR was performed using Fast SYBRTM Green Master Mix (Applied Biosystems) in a 7500 Fast Real‐Time PCR System (Applied Biosystems). The following primers were used for *Cd274* (FW: 5′ TGCGGACTACAAGCGAATCA and REV: 5′ GCTGGATCCACGGAAATTC) and β‐actin detection (FW: 5′ GGCTCCTAGCACCATGAAGA and REV: 5′ CCACCGATCCACACAGAGTA).

### 2.12 Human study and tissue

Selection of human ER^+^ BC tissue samples has been described previously [23]. Briefly, women with a histological diagnosis of hormone receptor positive BC, for whom tissue from a distant metastasis and full medical records were available, were eligible. Patients with synchronous metastases were excluded. The study protocol was approved by the Institutional Review Board of Hospital 12 de Octubre (‘Comité Ético de Investigación Clínica—Hospital 12 de Octubre’, Madrid, Spain; Study code: 11/137) and conducted according to the principles expressed in the Declaration of Helsinki. This review board waived the need for consent since all the samples belonged to patients diagnosed of cancer before 2007. According to the Royal Act in Biomedical Research in force in Spain since 2007 (Royal Act 14/2007, July 3), the retrospective collection of archival samples belonging to patients diagnosed before 2007 does not require individual signed informed consent.

### 2.13 Immunohistochemistry

For histological analyses, tissues were fixed in 10% buffered formalin (Sigma‐Aldrich) and embedded in paraffin. Immunohistochemical staining with anti‐PD‐L1 antibody (rabbit monoclonal antibody (E1L3N); Cell Signaling #13684) was performed on 2.5‐μm tissue sections. Immunohistochemistry was performed using an automated protocol developed for the Autostainer Link automated slide staining system (DAKO, Agilent, Santa Clara, CA, USA). All steps were performed on this staining platform using validated reagents, including deparaffinization, antigen retrieval (cell conditioning), and antibody incubation and detection. Corresponding stainings were acquired and digitalized using the AxioScan.Z1 system (Zeiss). Digitalized images were automatically analyzed with the axiovision version 4.6.2 software (Zeiss). The percentage of PD‐L1 positivity was considered as ratio of PD‐L1‐positive cells to total number of cells.

### 2.14 SA‐β galactosidase assay

Senescence was induced in MCF7 cells by treatment with 5 μm Nutlin‐3 (Selleckchem, M300F‐500) for indicated number of days. Senescent cells were stained for β‐galactosidase activity at pH 6 (CST, 9860).

### 2.15 Statistics

Statistical parameters and tests are reported in the figures and corresponding figure legends. Statistical analysis was done using graphpad prism version 8.0 (GraphPad Software Inc.). One‐way‐ANOVA was performed for all the datasets that required comparison among multiple data points within a given experimental condition.

## 3 Results

### 3.1 Modulation of PD‐L1 expression by medically approved drugs

**Fig. 1:**
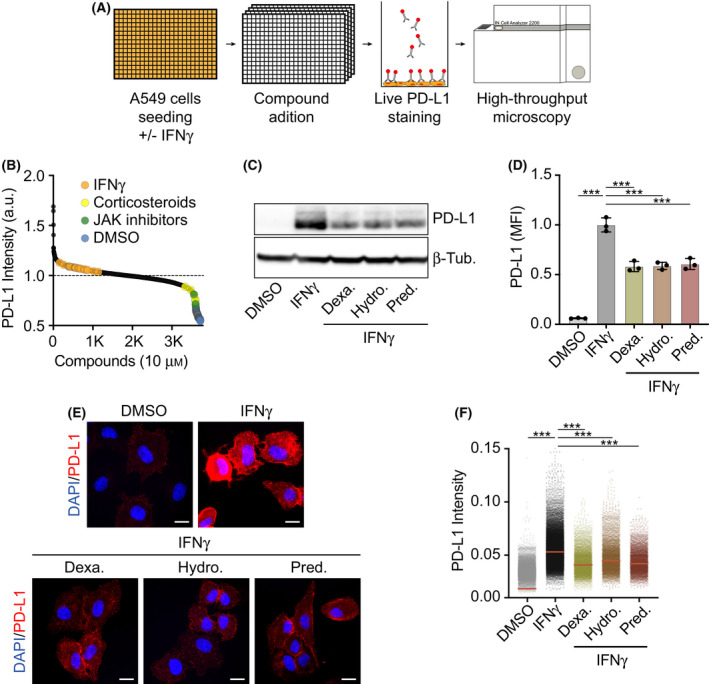
Evaluating the effect of medically approved drugs on IFN‐γ‐induced PD‐L1 expression. (A) Overview of the phenotypic screen workflow. Briefly, A549 cells were seeded in 100 ng·mL^−1^ IFN‐γ 24 h before addition of 4216 compounds at 10 μm. After 24 h of compound exposure, cells were stained with an anti‐PD‐L1 antibody conjugated to phycoerythrin and fixed with formaldehyde. Nuclei were stained with Hoechst, and the immunofluorescences were analyzed by HTM. (B) Hit distribution of the screen described in (A) illustrating the enrichment of JAK inhibitors and corticosteroids among the compounds reducing PD‐L1 signal in HTM. PD‐L1 levels in wells with only IFN‐γ and negative controls (DMSO) are also shown to illustrate the window of the assay. (C) Western blot illustrating the levels of PD‐L1 in A549 cells grown in the presence of DMSO as control or 100 ng·mL^−1^ IFN‐γ 24 h before addition of hydrocortisone (10 μm), prednisolone (10 μm), or dexamethasone (10 μm), for 24 h. β‐Tubulin levels are shown for loading control. (D) Quantification of flow cytometry‐mediated assessment of surface PD‐L1 levels in A549 cells after 24 h of control or compound exposure (treated as in (C)). Mean Fluorescence Intensity (MFI) values are relative to those observed in the control. One‐way ANOVA (*n* = 3) was used for statistical analysis ***P < 0.001, error bars indicate ± SD. (E) Representative immunofluorescence images of PD‐L1 (red) in A549 cells cultured in the presence or absence of IFN‐γ and the indicated compounds (treated as in (C)), nuclei are shown in blue. Scale bar (white), 5 μm. (F) HTM‐based quantification of PD‐L1 levels in A549 cells treated as in (C). One‐way ANOVA test (*n* = 3) was used to calculate statistical significance of the differences between groups, ***P < 0.001. All datapoints represent single‐cell measurements, with the horizontal red line indicating the median.

PD‐L1 levels in tumor biopsies are one of the biomarkers that have been shown to predict the response to cancer immunotherapy using anti‐PD‐L1 inhibitors [24]. In this context, we seek to determine how all medically approved drugs influence the surface expression of PD‐L1. To do so, we conducted a High‐Throughput screen using a library of 4216 compounds including 1200 FDA‐approved drugs and other chemicals at various stages of clinical development (Fig. 1A; see Materials and methods for details). The screening was conducted in the human lung cancer cell line A549, which was previously shown to express PD‐L1 upon IFN‐γ stimulation [25]. Since we were primarily focused on identifying downregulators of PD‐L1 expression, which could limit the efficacy of anti‐PD‐1/PD‐L1 therapies, the screening was conducted on A549 cells that were previously treated with 100 ng·mL^−1^ of IFN‐γ for 24 h and then subsequently with the compounds from the library for another 24 h. At this point, cells were stained with anti‐PD‐L1 antibodies, fixed, and processed for high‐throughput microscopy (HTM). As expected, wells treated with only IFN‐γ (positive control) showed a significant increase in PD‐L1 expression when compared to DMSO‐treated wells.

After analyzing the results from the screen, corticosteroids were the most enriched compound class among those lowering PD‐L1 expression (Fig. 1B and Table S1). Subsequent validation experiments using WB, flow cytometry, and high‐content microscopy confirmed that three independent corticosteroids (dexamethasone, hydrocortisone, and prednisolone) significantly reduced surface levels of PD‐L1 in IFN‐γ‐treated A549 cells (Fig. 1C–F). Besides corticosteroids, inhibitors of the Janus kinases (JAK1/2) were also found among the top downregulators, which is in agreement with their known role in the IFN‐γ‐dependent induction of PD‐L1 [26]. In addition to validating the usefulness of our approach, our findings help to understand why *JAK1/2* mutations [27] or a baseline corticosteroid treatment [28, 29] confer resistance to anti‐PD‐L1 therapy.

### 3.2 ERα signaling suppresses PD‐L1 expression in ER^+^ BC cells

In contrast to molecules lowering PD‐L1 expression, there were very few chemicals capable of substantially inducing PD‐L1 beyond the levels observed upon IFN‐γ treatment, which failed to be confirmed in subsequent validation experiments (many of the hits were related to autofluorescence of the compounds). We were nevertheless intrigued by the presence of the SERD fulvestrant among the top compounds from this list (Table S1). Of note, even if the chemical screening was done in A549 cells, these cells express ERα and fulvestrant reduces the growth of A549 xenografts [30]. In any case, and to further investigate the potential effect of antiestrogen therapies on increasing PD‐L1 expression we switched to MCF7, which is a widely used ER^+^ BC cell line.

**Fig. 2:**
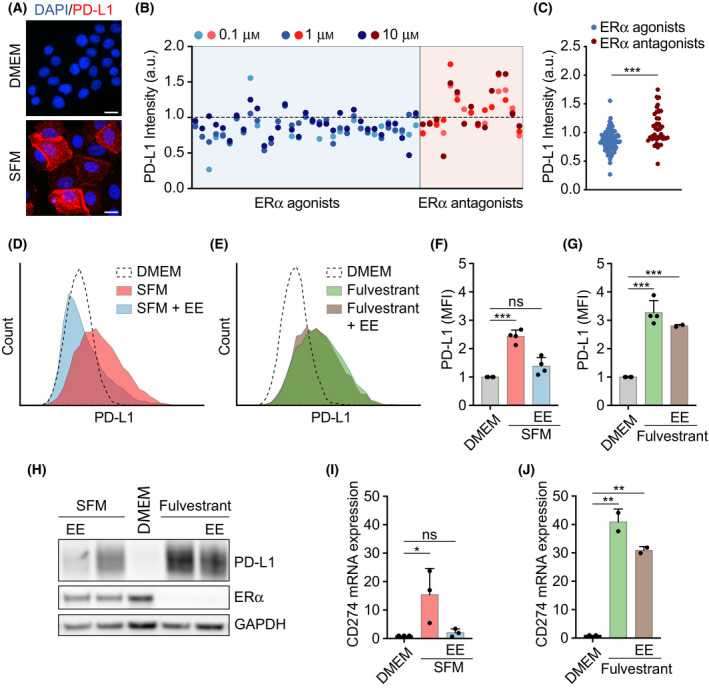
Estrogen‐dependent suppression of PD‐L1 expression in ER^+^ BC cells. (A) Immunofluorescence of PD‐L1 (red) in MCF7 cells cultured in normal or steroid‐free medium (SFM) for 15 days. DAPI (blue) was used to stain DNA. Representative images are shown. Scale bar (white), 5 μm. (B) Scatterplot of PD‐L1 intensity levels in SFM‐grown MCF7 cells treated with ERα agonists or antagonists, screened at three concentrations, 0.1, 1.0, and 10 µm. (C) Grouped comparison of PD‐L1 levels in response to ERα agonists and ERα antagonists from the experiment shown in (B) using a two‐tailed Student *t*‐test. (D, E) Flow cytometry‐mediated assessment of surface PD‐L1 levels in MCF7 cells grown in DMEM, SFM (D), or DMEM containing 1 μm fulvestrant (Fulv) (E) for 14 days. Where indicated, EE (10 nm) was added for the final 3 days. (F,G) Quantification of 4 independent flow cytometric experiments as shown in (D, E). Mean fluorescent intensity (MFI) values are relative to those observed in the control. Data represent the mean ± SD, ***P < 0.001 calculated by one‐way ANOVA. (H) Western blot illustrating the levels of PD‐L1 and ERα of MCF7 cells cultured as in (D, E). GAPDH levels are shown for loading control. (I, J) qRT‐PCR analysis of PD‐L1 (*CD274*) expression in MCF7 cells cultured in DMEM and SFM (*n* = 3 in I) or 1 μm fulvestrant (*n* = 2 in J) for 18 days. Where indicated, media were supplemented with EE (10 nm) for the last 3 days. *18S* RNA served as an internal control. Data represent the mean ± SD, statistical significance was determined by one‐way ANOVA. *P < 0.05; **P < 0.01; ***P < 0.001.

First, and in order to evaluate the effect of hormone deprivation on PD‐L1 expression, we grew MCF7 cells in SFM for two weeks. This led to a clear upregulation of PD‐L1, which was present on the cell membrane (Fig. 2A). Using this experimental setup, we conducted a focused chemical screen, where we tested the effects of 25 ER agonists and 11 ER antagonists in a dose–response (Fig. 2B, Table S2). Despite variability on the effects observed with individual compounds, there was a clear overall trend in that ER agonists reduced and antagonists increased PD‐L1 expression in SFM‐grown MCF7 cells (Fig. 2B,C). Flow cytometry data confirmed that either growing MCF7 cells on SFM or treating them with fulvestrant for two weeks led to a clear upregulation of surface PD‐L1 levels (Fig. 2D–G). Similar results were observed by WB (Fig. 2H). It is noteworthy that while a treatment with the synthetic estrogen ethinylestradiol (EE) downregulated PD‐L1 in SFM‐grown cells, it failed to do so in those treated with fulvestrant, which is explained by the fact that these cells lack ERα expression (Fig. 2H). Likewise, even if SFM or fulvestrant induced PD‐L1 expression in several ERα^+^ cell lines, this effect was not seen in ERα^−^ ones (Fig. S1). Quantitative reverse transcription–polymerase chain reaction (qRT‐PCR) analyses revealed that the upregulation of PD‐L1 (*CD274*) levels in SFM‐ or fulvestrant‐treated MCF7 cells occurred at the level of transcription (Fig. 2I,J). Finally, RNA interference‐mediated downregulation of ERα (*ESR1*) but not ERβ (*ESR2*) also led to the upregulation of transcription and surface PD‐L1 levels in MCF7 cells (Fig. S2). Collectively, these experiments reveal that estrogens suppress PD‐L1 expression in ER^+^ BC cells through the stimulation of ERα signaling.

### 3.3 ERα inversely correlates with PD‐L1 expression in breast cancer

Recent analyses of The Cancer Genome Atlas project have indicated higher levels of PD‐L1 in triple‐negative BC (TNBC) when compared to non‐TNBC subtypes [31]. Based on our findings, we explored whether this correlation could be linked to the expression of ERα. Indeed, gene expression analysis of 1904 BCs (ERα^+^: 1459; ERα^−^:445) from the METABRIC cohort of the European Genome‐Phenome Archive (EGA) dataset [21] revealed significantly higher levels of PD‐L1 mRNA expression (*CD274*) in the ERα^−^ cohort (Fig. S3A). Similar results could be observed at the protein level when comparing PD‐L1 expression between 5 ERα^+^ cell lines (MCF7, T47D, CAMA‐1, ZR‐75‐1 and BT‐474) and 3 ERα^−^ ones (MDA‐MB‐231, HCC1937 and BT‐549; Fig. S3B).

Next, to determine whether PD‐L1 expression was also induced *in vivo* in response to an antiestrogen treatment, we used a transgenic mouse model of ER^+^ BC based on the expression of the Polyoma Virus middle T Antigen downstream of the mouse mammary tumor virus long terminal repeat (MMTV‐PyMT) [32]. In agreement with our *in vitro* findings, treatment of MMTV‐PyMT transgenic mice harboring BC with the SERM tamoxifen led to a significant increase in PD‐L1 expression in the tumors (Fig. S3C,D). Finally, we evaluated PD‐L1 expression in tissue from a small cohort of human patients of ER^+^ BC (clinical and demographic characteristics detailed in Table S3) for which we obtained paired biopsies from the primary tumor and the metastases that emerged during or after adjuvant hormonal therapy. While PD‐L1 expression was virtually absent in the primary tumors, areas of PD‐L1‐expressing cells could be detected in half of the metastatic samples (Fig. S3E,F).

### 3.4 Estrogen deprivation activates a broad immune‐suppressive transcriptional program in MCF7 cells

**Fig. 3:**
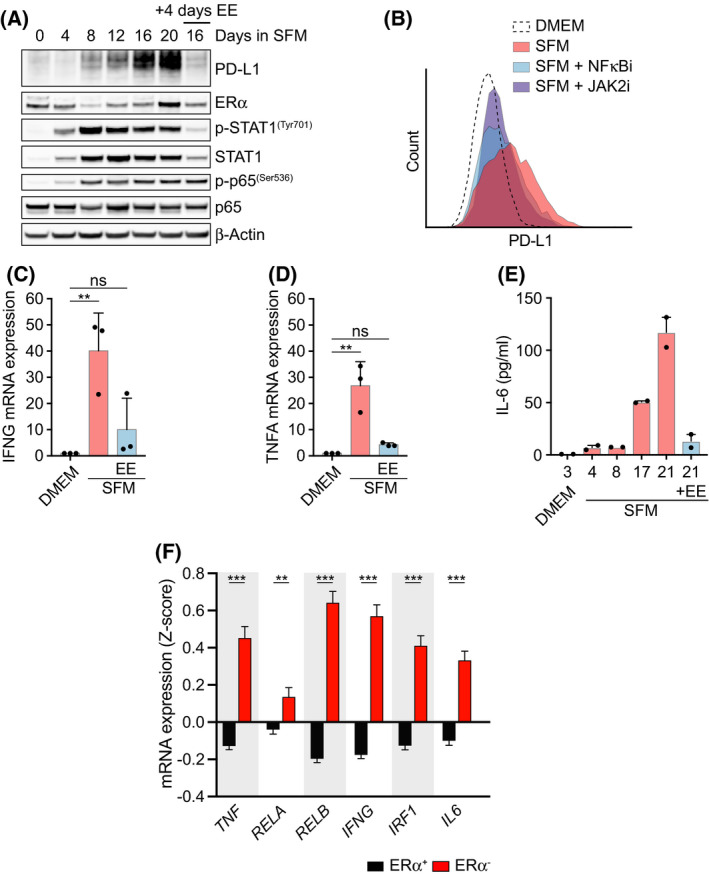
Estrogen signaling suppresses an inflammatory phenotype in MCF7 cells. (A) Whole‐cell lysates from MCF7 cells cultured in SFM for the specified days were analyzed by WB using the indicated antibodies. Where indicated, 10 nm EE was added at 16 days for the final 4 days. Total p65 and β‐Actin served as loading controls. (B) Flow cytometry‐mediated evaluation of PD‐L1 membrane levels in MCF7 cells grown in DMEM or SFM for 21 days, alone or in combination with the NF‐kB inhibitor CAPE (10 μm) or JAK2 inhibitor CEP‐33779 (10 μm) for the last 3 days. Representative data from 3 experiments are shown. (C, D) qRT‐PCR analysis (*n* = 3) of IFN‐γ (*IFNG*) (C) or TNF‐α (*TNFA*) (D) mRNA levels in MCF7 cells cultured as in (A). *18S* rRNA was used as an internal control. (E) Levels of IL‐6 in the supernatant of MCF7 cells cultured in DMEM or SFM for the specified days as measured in duplicates by LEGENDplex‐FACS (see Materials and methods). Where indicated, EE (10 nm) was added at day 17 for the last 4 days. (F) *TNF*, *RELA*, *RELB*, *IFNG, IRF1*, and *IL6* mRNA expression levels in ERα^+^ (*n* = 1459) and ERα^−^ (*n* = 445) patient samples. Normalized *Z*‐scores were extracted from the METABRIC dataset [22]. Data are presented as mean ± SEM, and two‐tailed Student's *t*‐test was used to calculate the statistical significance. **P < 0.01 and ***P < 0.001.

To determine the mechanism by which estrogen deprivation induces PD‐L1, we first analyzed how it impacts on the activity of JAK‐signal transducer and activator of transcription proteins (JAK‐STAT) and NF‐κB signaling pathways, both of which are key regulators of PD‐L1 expression [27, 33, 34, 35]. In fact, a time course of MCF7 cells grown in SFM revealed that both pathways were activated, as evidenced by the phosphorylation of STAT1 at Tyr 701 (p‐STAT1^Tyr701^) and RelA (p65) at Ser 536 (p‐p65^Ser536^), concomitantly to the upregulation of PD‐L1 (Fig. 3A). Moreover, treatment with a JAK2 inhibitor (CEP‐33779) or the NF‐κB inhibitor [caffeic acid phenethyl ester 9 (CAPE)] reduced the upregulation of PD‐L1 induced by SFM in MCF7 cells (Fig. 3B). Addition of EE after 16 days in SFM for 4 days reverted STAT1 but not p65 phosphorylation, arguing that activation of the JAK/STAT pathway rather than NF‐κB is the primary mediator of upregulating PD‐L1 in response to estrogen deprivation (Fig. 3A).

As to how JAK/STAT and NF‐κB signaling are activated upon estrogen deprivation, we found increased mRNA levels of IFN‐γ and tumor necrosis factor alpha (TNF‐α) in SFM‐grown MCF7 cells, which are the primary cytokines involved in the activation of each pathway, respectively (Fig. 3C,D). Interestingly, and besides IFN‐γ, estrogen deprivation also induced the secretion of IL‐6, which is a central inflammatory cytokine that stimulates JAK/STAT signaling and that is known to decrease the effectiveness of cancer immunotherapy (Fig. 3E) [36]. Consistent with these *in vitro* findings, analysis of the METABRIC dataset containing transcriptomic analyses of 1904 BC patients revealed significantly higher levels of *TNF*, *RELA, RELB, IFNG, IRF1*, *and IL6* mRNA expression in ERα^−^ tumors when compared to ERα^+^ ones (Fig. 3F).

**Fig. 4:**
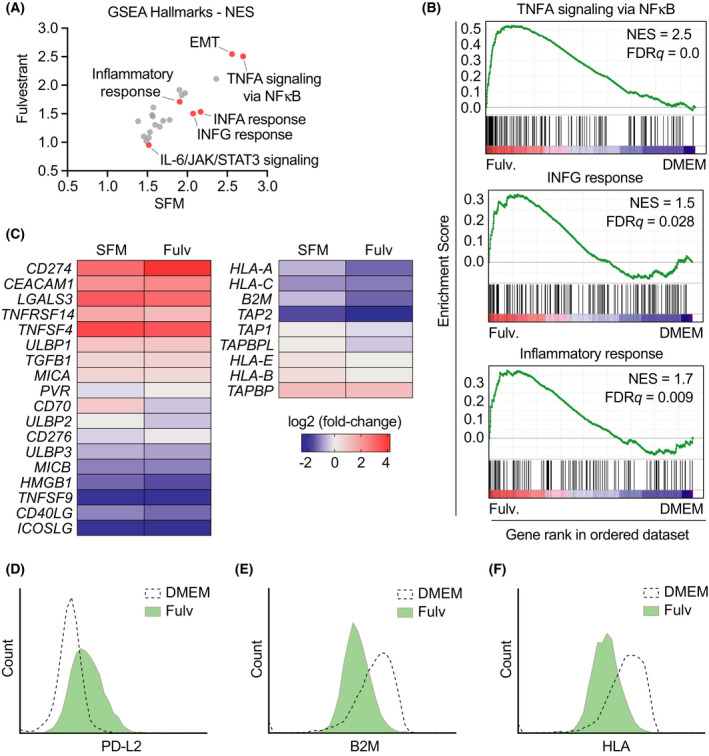
Estrogen deprivation drives expression of immune checkpoints together with silencing of the antigen‐presenting machinery. (A) GSEA hallmark gene sets ranked by normalized enrichment score (NES) comparing the transcriptional programs triggered by 3‐week treatments with fulvestrant (1 μm) or SFM in MCF7 cells, both normalized to DMEM (*n* = 3 biological replicates). Selected hallmarks are indicated in red. (B) Preranked GSEA on the genes from the hallmarks ‘TNFA signaling via NF‐κB’, ‘IFNG response’, and ‘inflammatory response’ obtained from RNAseq analysis comparing the transcriptome of MCF7 cells grown in DMEM or fulvestrant (1 μm) for 3 weeks. The heatmap representation illustrates the overall upregulation of these pathways in estrogen‐deprived MCF7 cells. (C) Representation of the expression of selected mRNAs related to immune checkpoints (left) and antigen‐presenting machinery (right), comparing the levels in SFM‐ or fulvestrant‐treated cells vs DMEM (as in (A)). (D–F) Flow cytometry‐mediated assessment of PD‐L2 (D), B2M (E), and HLA (F) levels in MCF7 cells grown in DMEM or fulvestrant (1 μm) for 14 days. Note that the antibody that was used for HLA detects 3 isoforms (HLA‐A, HLA‐B, and HLA‐C). Representative results out of three experiments are shown.

To obtain a general view of the transcriptional changes induced by estrogen deprivation in ER^+^ BC cells, we conducted RNA sequencing (RNAseq) in MCF7 cells grown in SFM or with fulvestrant for 3 weeks. We should note that while previous works have analyzed the effect of estrogen signaling on the transcriptome, these studies were focused on short‐term treatments aiming to the discovery of direct targets of ERα, days before we observe the induction of PD‐L1 expression or the activation of JAK/STAT and NF‐kB pathways [37, 38]. Analysis of GSEA hallmarks showed a good correlation between the transcriptional changes induced by both conditions (Fig. 4A). One of the common hallmarks was the epithelial–mesenchymal transition (EMT), which is consistent with the change in morphology that is observed with these treatments. Moreover, and in support to the previous data, ‘TNF‐α signaling via NF‐κB’, ‘IFN‐γ response’, or ‘inflammatory response’ was among the most significantly induced hallmarks (Fig. 4B and Fig. S4).

Interestingly, a specific analysis of immune‐related genes revealed that prolonged estrogen deprivation triggered the expression of multiple immune checkpoints besides PD‐L1 such as *CEACAM1*, *MICA*, or *LGALS2*, which was concomitant to a generalized suppression of the antigen‐presenting machinery, including reduced expression of *HLA‐A, HLA‐C*, *B2M*, and *TAP2* (Fig. 4C). Flow cytometry confirmed the upregulation of additional immune checkpoints such as PD‐L2 and reduced levels of beta‐2‐microglobulin (B2M) and HLA‐A in MCF7 cells treated with fulvestrant (Fig. 4D–F). Equivalent results were obtained in another ER^+^ BC cell line, T47D, both by FACS and by qRT‐PCR (Fig. S5A–G). Furthermore, analysis of transcriptomic data from the METABRIC cohort revealed a generalized increase in expression of multiple immune checkpoints in ER+ BC patients undergoing hormone therapy (Fig. S5H) [22, 23]. Collectively, these analyses reveal that persistent inhibition of estrogen signaling triggers a broad immunosuppressive transcriptional program in MCF7 cells.

### 3.5 Estrogen deprivation‐induced growth arrest is necessary but not sufficient for the activation of the inflammatory program in ER^+^ BC cells

Given our observation that estrogen‐deprived MCF7 cells express IL‐6, which is an important component of the senescence‐associated secretory phenotype (SASP) [39], we wondered whether the activation of an inflammatory transcriptional program was part of a SASP response in these cells, which are indeed growth‐arrested. Consistent with this view, MCF7 cells grown in SFM showed several features of senescence such as increased levels of p21^Cip1^ and histone H3 lysine 9 trimethylation (H3K9me3) or an increased activity of the senescence‐associated beta galactosidase (SA‐βgal; Fig. S6A,B). However, MCF7 cells induced to undergo senescence upon treatment with the p53 activator Nutlin‐3 failed to upregulate PD‐L1, indicating that simply arresting the growth of ER^+^ BC cells is not sufficient to trigger the same transcriptional response as that induced by estrogen deprivation (Fig. S6C).

**Fig. 5:**
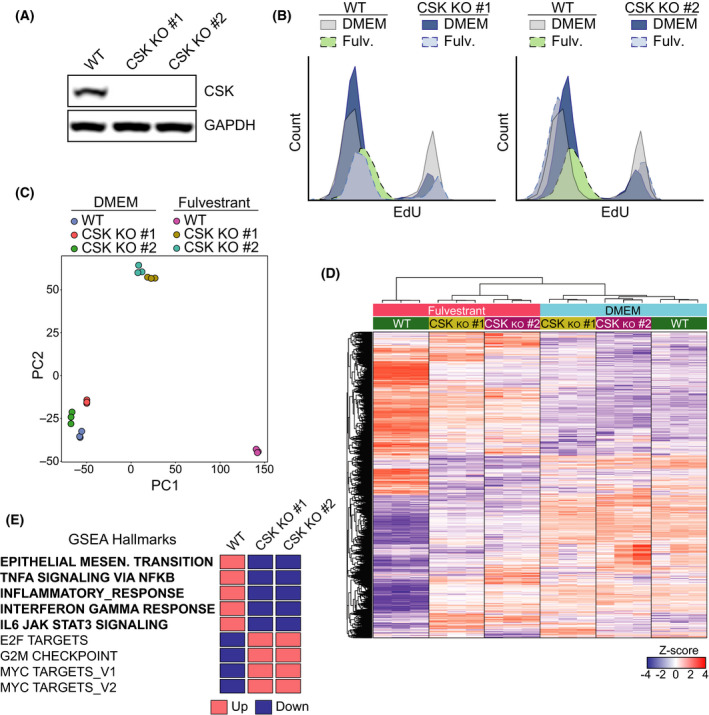
CSK‐dependent growth arrest mediates activation of the inflammatory phenotype in MCF7 cells. (A) Western blot illustrating the loss of CSK expression in 2 independent clones of CSK‐deficient MCF7 generated by CRISPR/Cas9. GAPDH is added as a loading control. (B) Flow cytometric data illustrating the growth arrest that is observed in WT MCF7 cells grown in fulvestrant for 20 days. Two clones of CSK‐deficient cells continue to incorporate EdU in the same conditions. (C) Principal component analysis (PCA) plot of an RNAseq experiment where the transcriptome of two independent MCF7 CSK^ko^ clones grown in DMEM with or without fulvestrant (1 μm) for 20 days was compared. The PCA plot was based on normalized gene counts after filtering for low‐expressed genes. (D) Heatmap and clustering of genes from the experiment defined in (C). The genes shown in the heatmap are genes that were found significantly regulated in any of the comparisons. Each gene (row) is standardized (*z*‐score) to mean = 0 and sd = 1 and then clustered by hierarchical clustering. Note that the fulvestrant‐induced changes in WT MCF7 cells are significantly milder in CSK‐deficient cells. (E) Impact of CSK deficiency on fulvestrant‐dependent expression of genes related to specific GSEA pathways from the experiment defined in (C). The full GSEA is provided in Table S4.

Nevertheless, and to further address whether the fulvestrant‐induced growth arrest contributes to the activation of inflammatory signaling, we deleted the CSK kinase in MCF7 cells using CRISPR, as this mutation has been shown to confer estrogen signaling‐independent growth on ER+ BC cells [40, 41]. As reported, CSK knockout (CSK^ko^) MCF7 cells continued to proliferate even in the presence of fulvestrant (Fig. 5A,B). We then conducted RNAseq on WT and CSK^ko^ MCF7 cells grown with or without fulvestrant for 3 weeks. Principal component analyses revealed that while the transcriptome of WT and CSK^ko^ MCF7 cells was similar in control conditions, the changes induced by fulvestrant were significantly attenuated in two independent clones of CSK‐deficient cells (Fig. 5C). A similar conclusion could be drawn from a heatmap illustrating the clustering of genes that were significantly regulated in this experiment (Fig. 5D). GSEAs identified biological pathways related to cell growth such as ‘E2F targets’, ‘G2M checkpoint’, or ‘MYC targets’ as those that were more significantly different between fulvestrant‐treated WT and CSK^ko^ MCF7 (Fig. 5E and Table S4). In contrast, the ‘epithelial–mesenchymal transition’ pathway, which, consistent with our previous analysis, was induced by fulvestrant in WT cells, was less so in CSK^ko^ MCF7 cells. Most importantly, the fulvestrant‐dependent induction of pathways such as ‘TNFA signaling via NF‐KB’, ‘inflammatory response’, ‘IL‐6 JAK/STAT3 signaling’, and ‘IFN‐γ response’ was reduced in CSK^ko^ cells. Together, these results indicate that the growth arrest triggered by estrogen deprivation is a necessary step for the subsequent activation of the immunosuppressive transcriptional program in ER+ BC cells. However, just arresting the growth of MCF7 cells is not sufficient to trigger this phenotypic change and the concomitant inhibition of estrogen signaling is necessary.

### 3.6 Estrogen deprivation limits T‐cell‐mediated cell killing of MCF7 cells independently of PD‐L1

**Fig. 6:**
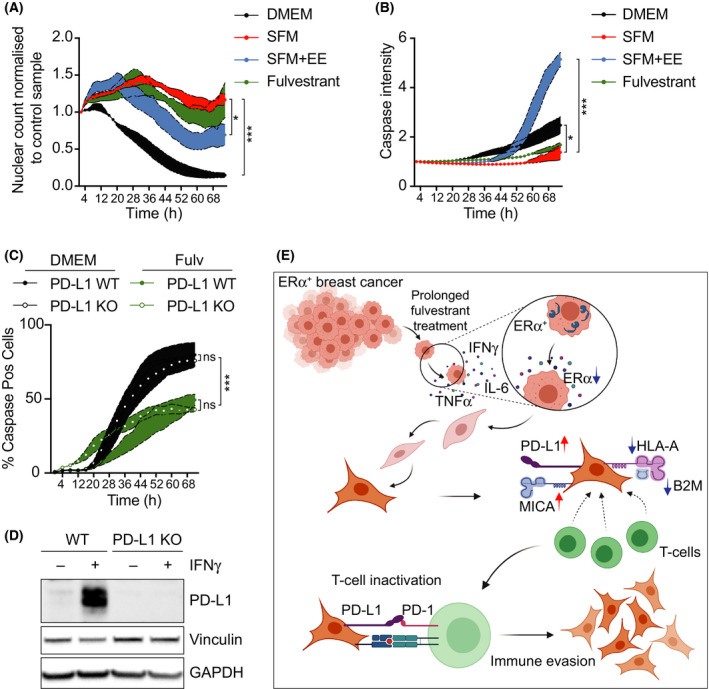
Estrogen signaling inhibition limits T‐cell‐mediated killing of MCF7 cells. (A) Live cell imaging of nuclei count from MCF7 cells after addition of activated primary T lymphocytes isolated from human peripheral blood. Nuclei counts were normalized to the value at time = 0 h for each condition, and then, each timepoint was subsequently normalized to the value of the control to which no T cells were added. (B) Time‐lapse microscopy of the intensity of a fluorescently labeled caspase‐3/7 substrate in MCF7 cells exposed to activated primary T cells. (C) Time‐lapse microscopy of the intensity of a fluorescently labeled caspase‐3/7 substrate in wild‐type (WT) or PD‐L1‐deficient (PD‐L1 KO) MCF7 cells exposed to activated primary T cells. (A–C) For these experiments, indicated cells were grown in normal media, SFM or fulvestrant (1 μm) for 2 weeks, prior to the addition of the activated T cells. Where indicated, EE (10 nm) was added for the last 3 days. One‐way ANOVA for (A, B) and two‐way ANOVA for (C) analyses were used to calculate the statistical significance of the differences between groups. All datapoints indicate mean values (*n* = 3) ± SEM (colored boundary). *P < 0.05 and ***P < 0.001. (D) Western blot illustrating the levels of PD‐L1 in WT and PD‐L1‐deficient MCF7 cells used in (C). Cells were treated with IFN‐γ to stimulate PD‐L1 expression. Vinculin and GAPDH levels are shown as loading controls. (E) Graphical summary of our work depicting that under prolonged hormone therapy, ER^+^ BC cells activate an inflammatory transcriptional program, which includes a generalized upregulation of immune checkpoint mediators together with the downregulation of the antigen‐presenting machinery. Hence, while hormone therapies efficiently arrest the growth of ER^+^ BC cells, they also promote a phenotype switch that favors their immune evasion.

Finally, we evaluated how estrogen deprivation in BC cells affected their sensitivity to being killed by immune cells, through a T‐cell‐mediated cell killing assay in MCF7 cells [42]. To do so, MCF7 cells stably expressing mCherry fused to a nuclear localization sequence were cocultured in the presence of activated primary T cells and followed by live cell imaging for 3 days. Remarkably, MCF7 cells that were previously grown in SFM or with fulvestrant were significantly resistant to their killing by T cells (Fig. 6A). In addition, EE was able to alleviate the effects of the SFM treatment and potentiated the elimination of MCF7 cells by T cells. Equivalent results were obtained by measuring apoptosis through the use of a fluorescent caspase‐3/7 target (Fig. 6B). Consistent with the transcriptomic data indicating that estrogen deprivation triggered multiple mechanisms of immunosuppression (including the upregulation of several immune checkpoints and downregulation of the antigen‐presenting machinery), PD‐L1 deficiency did not protect MCF7 cells from T‐cell killing, nor it modified the protection provided by fulvestrant (Fig. 6C,D). In summary, the experiments presented above revealed that, in addition to suppressing their growth, prolonged estrogen deprivation of ER^+^ BC cells promoted a phenotype switch that rendered them resistant to being killed by T cells through multiple independent immunosuppressive mechanisms (Fig. 6E).

## 4 Discussion

Our work started with the aim to identify how medically approved medicines influence the expression of PD‐L1, as PD‐L1 levels were previously shown to be potential biomarkers of efficacy of cancer immunotherapies using antibodies against PD‐1 or PD‐L1 [24]. We decided to conduct our screen in the presence of IFN‐γ, as this is the situation which we believe most represents the context in which PD‐L1 is actually expressed within tumors [43]. As expected, we were not able to find drugs that substantially increase PD‐L1 expression beyond what is induced by IFN‐γ, and our screen was mainly useful to identify drugs that can counteract this induction. Consistent with current knowledge, corticosteroids and JAK inhibitors were found to suppress PD‐L1 expression, which helps to understand their links to resistance to immunotherapy [27, 28, 29]. Besides these two classes of compounds, our manuscript provides a useful resource where investigators can evaluate how a given medicine affects PD‐L1 expression and could thus potentially affect the efficacy of cancer immunotherapies. As an example of this approach, and given its relevance for ER^+^ BC, we here explored in depth how the inhibition of estrogen signaling could be inducing PD‐L1 expression in cancer cells.

Estrogen was one of the first hormones to be described, and for decades, it was thought to only act in the female reproductive system [9]. Later studies revealed that estrogen receptors were widely expressed in many organs and that estrogens played pleotropic physiological roles beyond reproduction. Among these, several studies have indicated that estrogen levels influence the severity of diseases linked to inflammation, including cancer [11, 14, 44]. However, somewhat surprisingly, the link between estrogen signaling and inflammation has not been sufficiently addressed in the context of ER^+^ BC, which is the prototype tumor that is driven by estrogen signaling. Here, we show that estrogen deprivation triggers a broad immunosuppressive transcriptional program in ER^+^ BC cells, which includes the secretion of cytokines that activate NF‐κB signaling such as TNF‐α, but also additional cytokines such as IFN‐γ and IL‐6 that trigger the activation of the JAK/STAT pathway. As to how ER suppresses this inflammatory program, our work reveals that this is secondary to the persistent growth arrest that follows chronic inhibition of estrogen signaling in a manner that resembles the SASP secretory program that is observed in senescent cells. In this regard, to what extent senolytic compounds could help to eliminate the residual growth‐arrested BC cells that resist to the initial hormone therapy emerges as an interesting possibility to explore. Interestingly, a recent manuscript has revealed that acquired resistance to mitogen‐activated protein kinase inhibitors in melanoma also involves an EMT and the activation of an immunosuppressive transcriptional program that limits the efficacy of cancer immunotherapy, suggesting that this might be a recurrent phenomenon in cancer cells chronically treated with strategies that limit their growth [45].

Among the specific factors that are induced by chronic estrogen signaling deprivation, we found PD‐L1, which is consistent with a previous study that identified ERα as a direct transcriptional repressor of PD‐L1 [46]. Our study indicates that, while a direct regulation of the PD‐L1 promoter by ERα might exist, the main source of PD‐L1 expression upon estrogen deprivation is linked to the activation of JAK/STAT and NF‐κB signaling that occurs only after a prolonged treatment, more reminiscent of the clinical situation. Moreover, our study further demonstrates that the immunosuppressive phenotype of estrogen‐deprived ER^+^ BC cells is not just restricted to an upregulation of PD‐L1, but it includes the expression of multiple immune checkpoints together with a concomitant silencing of the antigen‐presenting machinery. Of note, and in addition to the activation of the inflammatory signals, sustained estrogen deprivation also triggers an EMT in MCF7 cells, which suggests that while the cancer cells might be growth‐arrested, their invasive properties might be unwantedly enhanced by endocrine therapy. BC has classically been considered as poorly responsive to immunotherapy due to initial failures in vaccination or cytokine treatments in the 1980s and 90s [47, 48], and since ER^+^ BC has a low mutational burden and is therefore immunologically ‘cold’. Our work here reveals yet another reason that could help to understand the limited efficacy of immunotherapies in BC, as while hormone therapy effectively arrests the growth of ER^+^ BC cells, it also triggers a broad immunosuppressive transcriptional program that limits their clearance by the immune system.

## 5 Conclusion

Hormone therapy is the current treatment for ER^+^ BC, the most frequent type of cancer in women worldwide. Unfortunately, immunotherapy has shown limited efficacy for the treatment of BC, even less so for ER+ tumors. We here report that chronic inhibition of ERα signaling triggers an immunosuppressive transcriptional program in ER^+^ BC cells, which includes the activation of multiple immune checkpoints such as PD‐L1 and PD‐L2, together with a reduced expression of the antigen‐presenting machinery. These findings indicate that, while hormone therapy succeeds in limiting the growth of ER^+^ tumors, treatment‐resistant cells acquire an immunosuppressive phenotype that hampers their elimination by the immune system.

## Conflict of interest

The authors declare no conflict of interest.

## Author contributions

DH and PM‐R contributed to most experiments and data analyses and to the preparation of the figures. CH contributed to the T‐cell killing experiments. SM and MQ‐F helped with experiments using MMTV‐PyMT animals and with human patient material. BP contributed to the experiments validating the effects of glucocorticoids and to the generation of PD‐L1‐deficient MCF7 cells. MH helped with the chemical screen. LL provided general technical support to many of the experiments. OF‐C and JC‐P supervised the study and wrote the MS.

### Peer review

The peer review history for this article is available at https://publons.com/publon/10.1002/1878‐0261.13083.

## Supporting information


**Fig. S1**. Estrogen‐deprival induces PD‐L1 expression in ER^+^ BC cells.
**Fig. S2**. ERα, and not ERβ, suppresses PD‐L1 expression in MCF7 cells.
**Fig. S3**. Inverse correlation between ERα and PD‐L1 in BC.
**Fig. S4**. Estrogen deprivation triggers an inflammatory transcriptional program in MCF7 cells.
**Fig. S5**. Estrogen‐deprival induces an inflammatory response in ERα^+^ T47D BC cells.
**Fig. S6**. Induction of senescence is not sufficient to trigger PD‐L1 expression in MCF7 cells.

**Table S1**. Results of the primary chemical screen in IFNγ‐stimulated A549 cells.

**Table S2**. Results of the ERα agonist/antagonist screen in MCF‐7 cells.

**Table S3**. Clinical and demographic characteristics of the ER^+^ BC patients used in Fig. S3.

**Table S4**. GSEA analysis comparing the transcriptomes of WT and CSK‐deficient MCF7 cells grown in fulvestrant or DMEM for 20 days.

## Data Availability

RNA sequencing data associated with this work are openly available from the GEO repository at https://www.ncbi.nlm.nih.gov/geo/, under accession numbers GSE134938 and GSE181909.
