# Supplementary material for: Prolonged estrogen deprivation triggers a broad immunosuppressive phenotype in breast cancer cells
Source: Mol Oncol. 2021 Aug 29;16(1):148–65. doi: 10.1002/1878-0261.13083 (PMC8732350; doi:10.1002/1878-0261.13083)
Supplement: Supplementary file 1 — Fig. S1. Estrogen‐deprival induces PD‐L1 expression in ER+ BC cells. Fig. S2. ERα, and not ERβ, suppresses PD‐L1 expression in MCF7 cells. Fig. S3. Inverse correlation between ERα and PD‐L1 in BC. Fig. S4. Estrogen deprivation triggers an inflammatory transcriptional program in MCF7 cells. Fig. S5. Estrogen‐deprival induces an inflammatory response in ERα+ T47D BC cells. Fig. S6. Induction of senescence is not sufficient to trigger PD‐L1 expression in MCF7 cells. [file MOL2-16-148-s002.pdf]

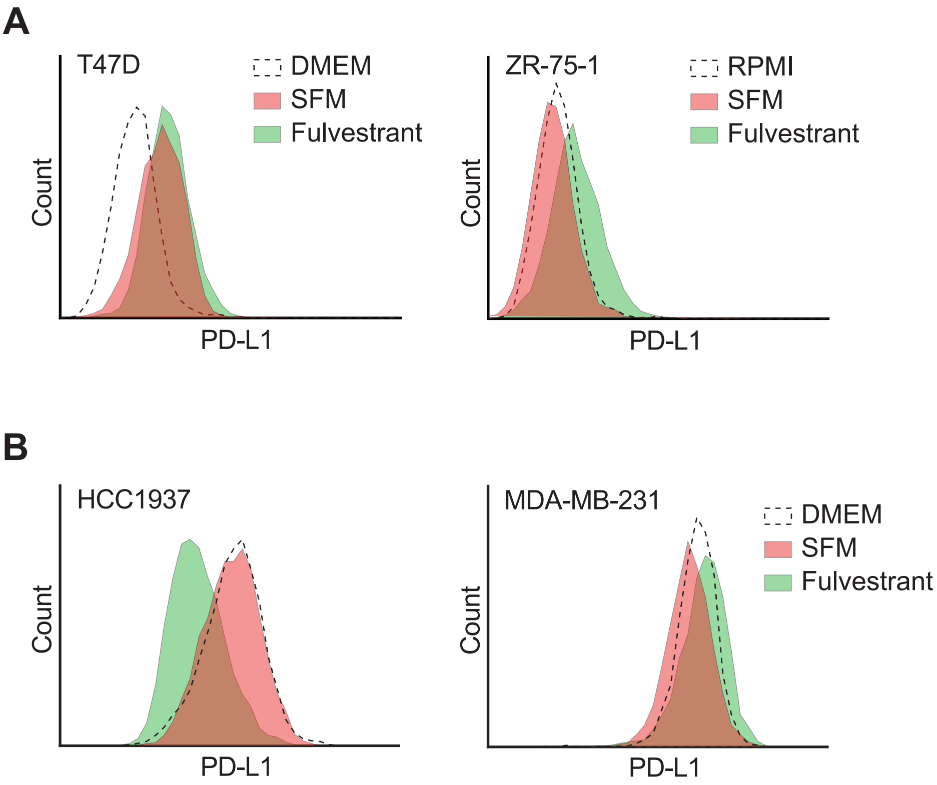
A
T47D
DMEM
SFM
Fulvestrant
Count
PD-L1
ZR-75-1
RPMI
SFM
Fulvestrant
Count
PD-L1
B
HCC1937
Count
PD-L1
MDA-MB-231
DMEM
SFM
Fulvestrant
Count
PD-L1

Figure S1. **Estrogen deprivation induces PD-L1 expression in ER+ BC cells.** (**A**) Flow cytometry-mediated quantification of PD-L1 surface levels in ER^+ BC cell lines, T47D and ZR-75-1, grown in DMEM, SFM or DMEM supplemented with fulvestrant (1 μM) for 18 days. (**B**) Flow cytometry-mediated quantification of PD-L1 surface levels in ER– BC cell lines (HCC1937 and MDA-MB-231), grown in normal medium (DMEM for MDA-MB-231 and RPMI 1640 for HCC1937), SFM or medium supplemented with fulvestrant (1 μM) for 18 days.

Hühn D *et al.* **Fig. S2**

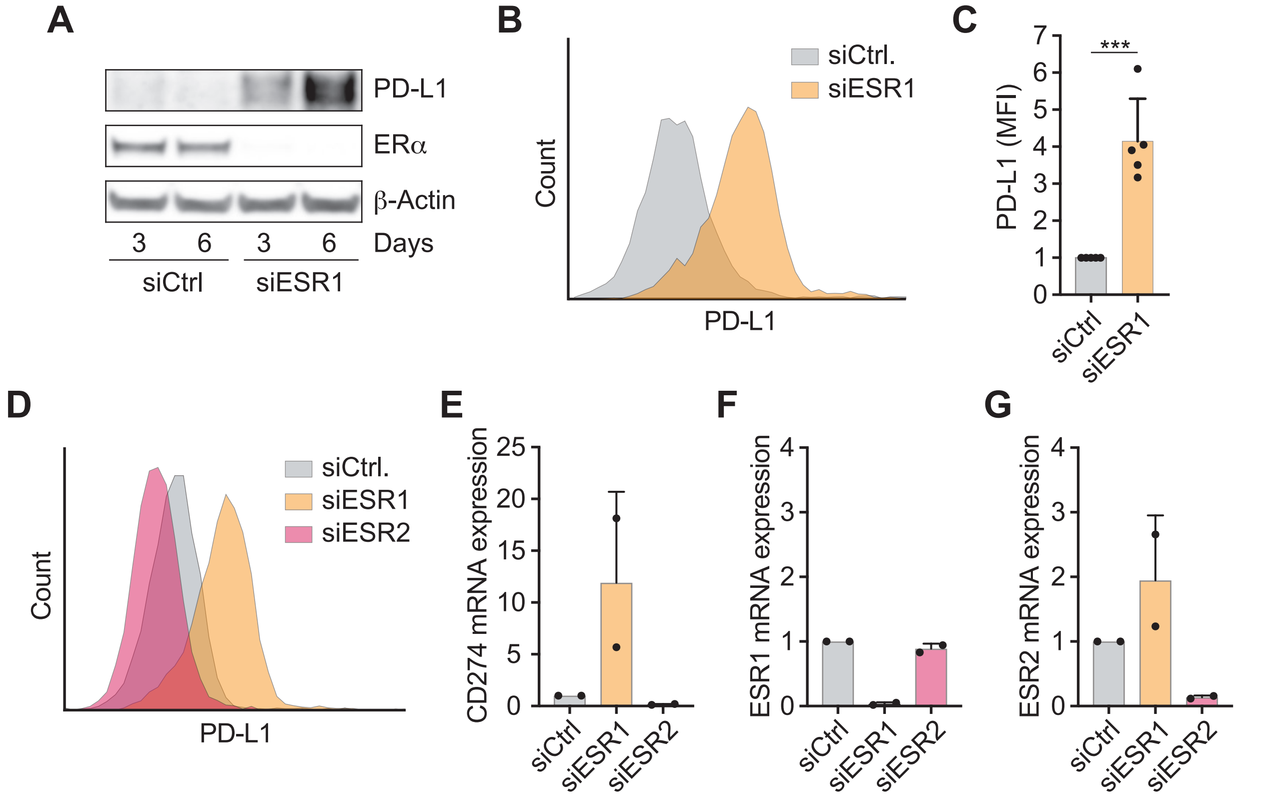

Figure S2. **ERα, and not ERβ, suppresses PD-L1 expression in MCF7 cells.** (**A**) Western blot analysis of the indicated proteins in MCF7 cells treated with either 20 nM control or ERα-targeting (*ESR1*) siRNA for 3 or 6 days. β-Actin is shown as a loading control. (**B**) PD-L1 membrane levels analysed by flow cytometry after treatment of MCF7 cells with the indicated siRNAs for 6 days. (**C**) Quantification of 3 independent experiments as the one shown in (**B**). Data are presented as mean +SD (n=5). ***p<0.001. (**D**) PD-L1 membrane levels as analysed by flow cytometry in MCF7 cells transfected twice with 20nM of the indicated siRNAs for 6 days. (**E-G**) qRT-PCR analysis of *CD274* (**E**), *ESR1* (**F**) and *ESR2* (**G**) mRNA levels in MCF7 cells transfected as in (**D**). Levels of the *18S* rRNA served as an internal control.

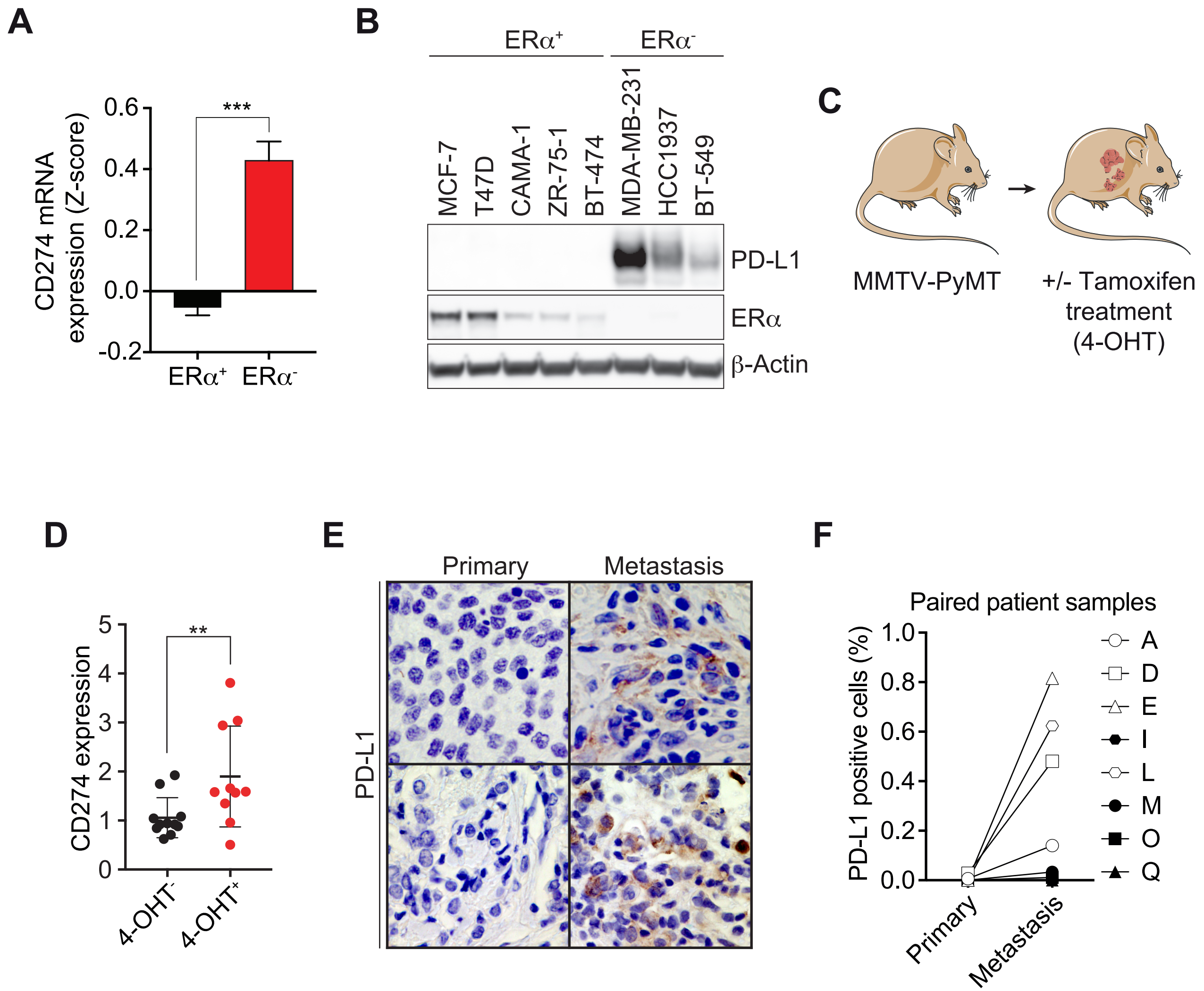
A
CD274 mRNA expression (Z-score)
***
0.6
0.4
0.2
0.0
-0.2
ERα+ ERα-
B
ERα+
ERα-
MCF-7
T47D
CAMA-1
ZR-75-1
BT-474
MDA-MB-231
HCC1937
BT-549
PD-L1
ERα
β-Actin
C
MMTV-PyMT
+/- Tamoxifen treatment (4-OHT)
D
CD274 expression
**
5
4
3
2
1
0
4-OHT-
4-OHT+
E
Primary
Metastasis
PD-L1
F
Paired patient samples
PD-L1 positive cells (%)
1.0
0.8
0.6
0.4
0.2
0.0
Primary
Metastasis
A
D
E
I
L
M
O
Q

Figure S3. **Inverse correlation between ERα and PD-L1 in BC.** (**A**) *CD274* mRNA expression levels in ERα$^+$ (n=1459) and ERα$^-$ (n=445) patient samples. Normalised Z-scores were extracted from the METABRIC breast cancer dataset. A factor was added to both populations in order to set the ERα+ group to 0. Data are presented as mean +SEM and two-tailed Student's t-test was used to calculate the statistical significance, ***p<0.001. (**B**) Western blot analysis of PD-L1 and ERα protein in a panel of ERα$^+$ and ERα$^-$ BC cell lines. β-Actin served as a loading control. (**C**) Schematics of the experimental procedure in mice. MMTV-PyVT mice that spontaneously develop mammary tumors were treated daily from week 5 until with 4-hydroxytamoxifen (4-OHT) or vehicle. (**D**) *Cd274* expression in tumors isolated from MMTV-PyVT mice treated with 4-OHT or vehicle as determined by RT-qPCR. Data were normalized to β-*Actin* mRNA levels. Graph represents the mean ±SD and two-tailed Student's t-test was used to determine statistical significance, **p<0.01. (**E**) Immunohistochemical staining of PD-L1 (brown) in paired samples of primary and metastatic human ER+ BC from two different patients. Sections were stained with Hematoxylin-Eosin to aid in the pathological analysis. (**F**) Quantification of the percentage of PD-L1 positive cells in paired primary tumor and metastasis samples from 8 patients of ERα-positive BC.

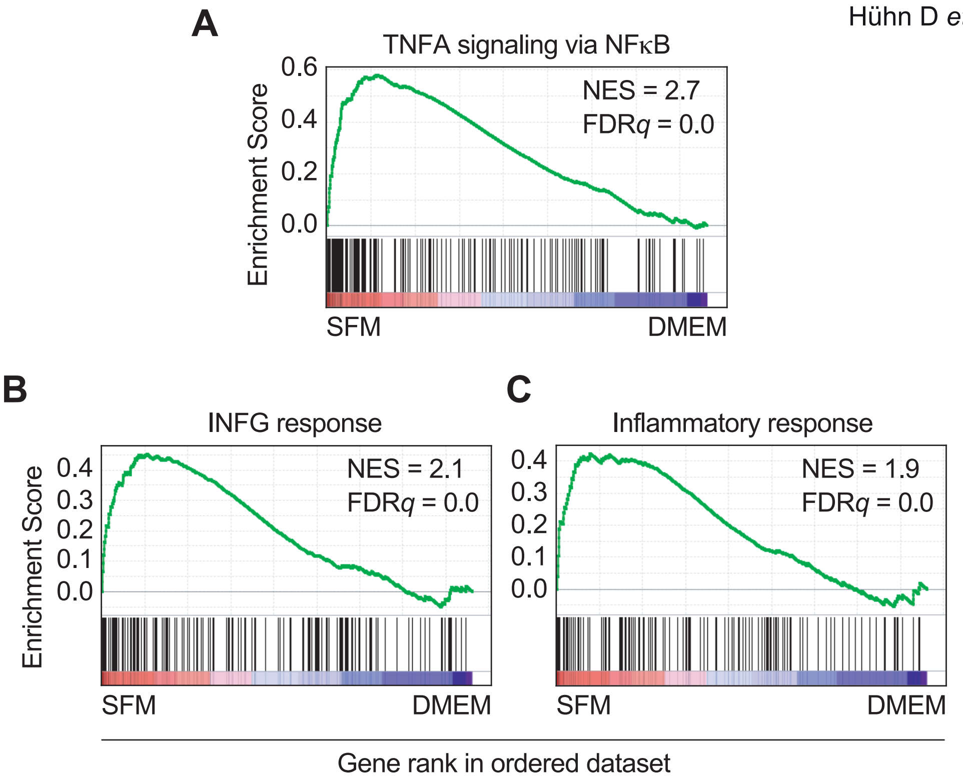
A
TNFA signaling via NFκB
Enrichment Score
0.6
0.4
0.2
0.0
NES = 2.7
FDRq = 0.0
SFM
DMEM
B
INFG response
Enrichment Score
0.4
0.3
0.2
0.1
0.0
NES = 2.1
FDRq = 0.0
SFM
DMEM
C
Inflammatory response
0.4
0.3
0.2
0.1
0.0
NES = 1.9
FDRq = 0.0
SFM
DMEM
Gene rank in ordered dataset

Figure S4. **Estrogen deprivation triggers an inflammatory transcriptional program in MCF7 cells.** (**A**-**C**) Pre-ranked GSEA on the genes from the hallmarks "TNFA signaling via NF-κB" (**A**), "IFNG response" (**B**) and "Inflammatory response" (**C**) obtained from RNAseq analysis comparing the transcriptome of MCF7 cells grown in DMEM or SFM for 3 weeks. The heatmap representation illustrates the overall upregulation of these pathways in SFM-grown MCF7 cells.

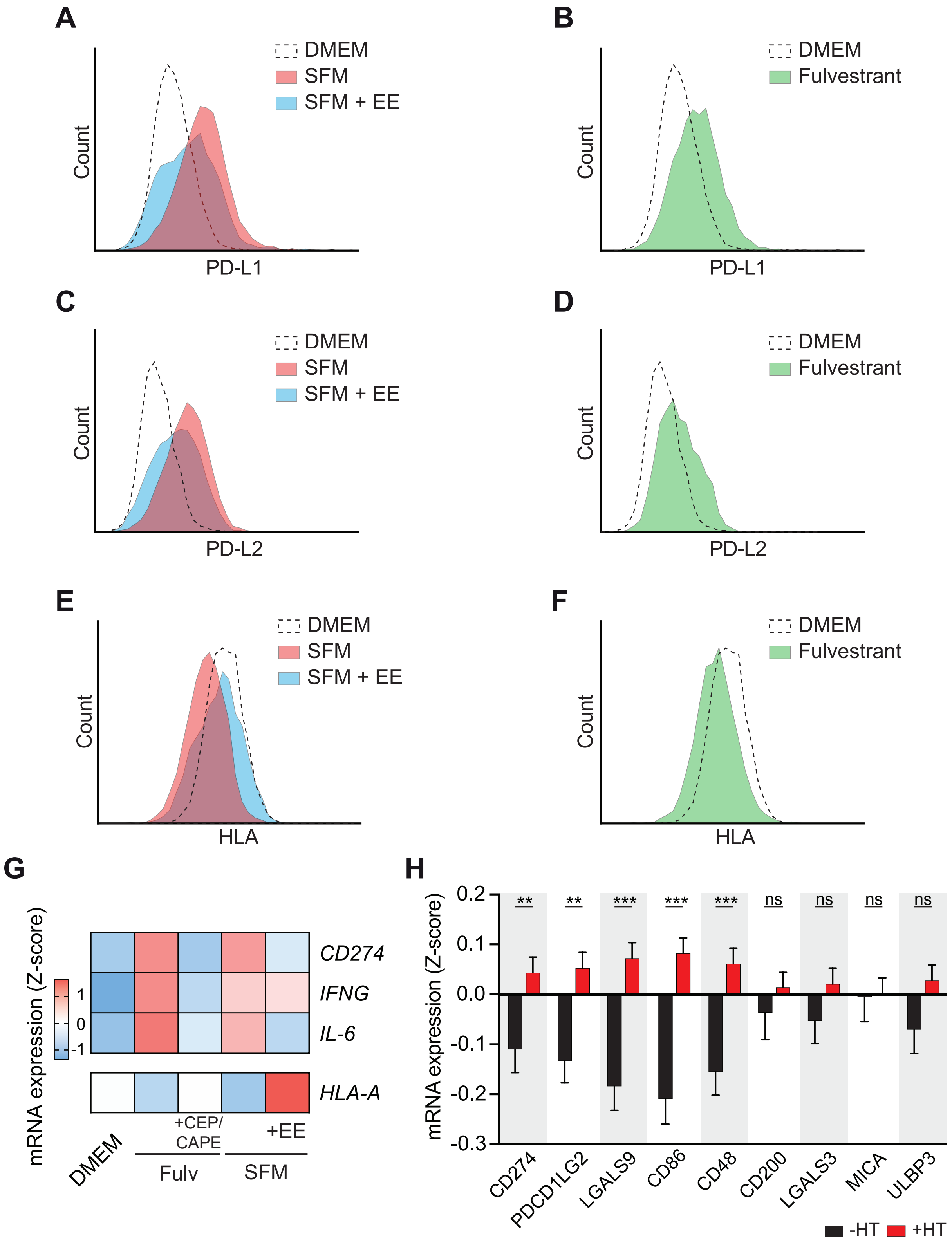
A
DMEM
SFM
SFM + EE
Count
PD-L1
B
DMEM
Fulvestrant
Count
PD-L1
C
DMEM
SFM
SFM + EE
Count
PD-L2
D
DMEM
Fulvestrant
Count
PD-L2
E
DMEM
SFM
SFM + EE
Count
HLA
F
DMEM
Fulvestrant
Count
HLA
G
mRNA expression (Z-score)
1
0
-1
CD274
IFNG
IL-6
HLA-A
DMEM
+CEP/
CAPE
+EE
Fulv
SFM
H
mRNA expression (Z-score)
0.2
0.1
0.0
-0.1
-0.2
-0.3
**
**
***
***
***
ns
ns
ns
ns
CD274
PDCD1LG2
LGALS9
CD86
CD48
CD200
LGALS3
MICA
ULBP3
-HT
+HT

Figure S5. **Estrogen deprivation induces the expression of immune checkpoints and the downregulation of HLA in T47D cells.** (**A, C, E**) Flow cytometry-mediated assessment of surface PD-L1 (**A**), PD-L2 (**C**) or HLA-A, -B, -C levels (**E**) in T47D cells grown in DMEM or SFM for 21 days. Where indicated EE (10nM) was added for the final 4 days. (**B, D, F**) Flow cytometry-mediated assessment of surface PD-L1 (**A**), PD-L2 (**C**) or HLA-A, -B, -C levels (**E**) in T47D cells grown in DMEM or DMEM supplemented with 1uM Fulvestrant for 21 days. (**G**) Heatmap of mRNA expression levels (Z-score) of the indicated genes as analyzed by qRT-PCR in T47D cells grown in DMEM or SFM for 21 days, alone or in combination with the NFkB inhibitor CAPE (10μM) or JAK2 inhibitor CEP-33779 (10μ) for the last 4 days. Where indicated EE (10nM) was added for the final 4 days. *18S* RNA served as an internal control. (**H**) mRNA expression levels of the indicated genes related to immune checkpoints in ERα^{+} patients receiving (+HT; 1047) or not receiving (-HT; 412) hormone therapy. Normalised Z-scores were extracted from the METABRIC breast cancer dataset. A factor was added to both populations in order to set the ERα+ group to 0. Data are presented as mean +SEM and two-tailed Student's t-test was used to calculate the statistical significance, **p<0,01;***p<0.001;ns: not significant.

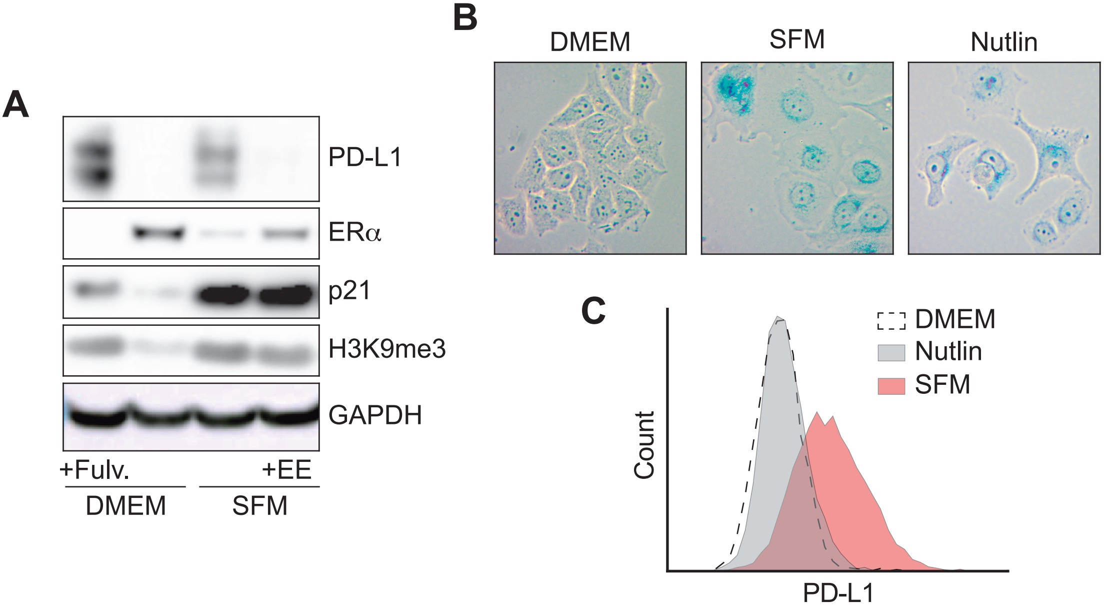
A
PD-L1
ERα
p21
H3K9me3
GAPDH
+Fulv.
+EE
DMEM
SFM
B
DMEM
SFM
Nutlin
C
DMEM
Nutlin
SFM
Count
PD-L1

Figure S6. **Induction of senescence is not sufficient to trigger PD-L1 expression in MCF7 cells.** (**A**) MCF7 cells were grown in DMEM, SFM or DMEM with fulvestrant (1 μM) for 17 days. Where indicated, EE (10 nM) was added for the last 3 days. Whole-cell extracts were subjected to western blot analysis of the indicated proteins. (**B**) SA-β-Galactosidase assay of MCF7 cells cultured in DMEM, SFM (19 days) or Nutlin-3 (5 μM, 5 days). Representative images for each condition are shown. (**C**) FACS analysis of PD-L1 membrane levels in MCF7 cells cultivated as in (**B**).
