# Supplementary material for: Prolonged estrogen deprivation triggers a broad immunosuppressive phenotype in breast cancer cells
Source: Mol Oncol. 2021 Aug 29;16(1):148–65. doi: 10.1002/1878-0261.13083 (PMC8732350; doi:10.1002/1878-0261.13083)
Supplement: Supplementary file 4 — Table S3. Clinical and demographic characteristics of the ER+ BC patients used in Fig. S3. [file MOL2-16-148-s001.docx]

**Table S3. Clinical and demographic characteristics**

**of the ER^+^ BC patients used in Fig. S3.**

| **Characteristic** | **N=8** |
| --- | --- |
| **Age (median, range)** | 53.6 (35.1-68.2) |
| **Tumor size**  T1  T2  T3  T4 | 0 (0%)  6 (75%)  1 (12.5%)  1 (12.5%) |
| **Nodal stage**  N0  N1 | 2 (25%)  6 (75%) |
| **Grade**  G1  G2  G3 | 0 (0%)  7 (87.5%)  1 (12.5%) |
| **Adjuvant hormonal treatment**  -Tamoxifen only  -Switch (Tamoxifen 🡪 Aromatase inhibitor)  -Aromatase inhibitor only | 0 (0%)  3 (37.5%)  4 (50%)  1 (12.5%) |
| **Disease-free interval (years: median, range)**  -Relapse during adjuvant hormonal therapy  -Relapse after completion of adjuvant hormonal therapy | 4.5 (2.84 – 15.1)  5 (62.5%)  3 (37.5%) |
| **Hormonal receptors**  ER and/or PR positive  HER2-positive | 8 (100%)  0 (0%) |
| **Baseline Ki67**  <15%  >14% | 5 (62.5%)  3 (37.5%) |
